# Transcriptional regulation of mouse alpha *A-crystallin *gene in a 148kb *Cryaa *BAC and its derivates

**DOI:** 10.1186/1471-213X-8-88

**Published:** 2008-09-19

**Authors:** Louise Wolf, Ying Yang, Eric Wawrousek, Ales Cvekl

**Affiliations:** 1 The Departments of Ophthalmology and Visual Sciences, Bronx, NY 10461, USA; 2 Molecular Genetics, Albert Einstein College of Medicine, Bronx, NY 10461, USA; 3 Laboratory of Molecular and Developmental Biology, National Eye Institute, National Institutes of Health, Bethesda, Maryland 20892, USA

## Abstract

**Background:**

αA-crystallin is highly expressed in the embryonic, neonatal and adult mouse lens. Previously, we identified two novel distal control regions, DCR1 and DCR3. DCR1 was required for transgenic expression of enhanced green fluorescent protein, EGFP, in lens epithelium, whereas DCR3 was active during "late" stages of lens primary fiber cell differentiation. However, the onset of transgenic EGFP expression was delayed by 12–24 hours, compared to the expression of the endogenous *Cryaa *gene.

**Results:**

Here, we used bacterial artificial chromosome (BAC) and standard transgenic approaches to examine temporal and spatial regulation of the mouse *Cryaa *gene. Two BAC transgenes, with EGFP insertions into the third coding exon of *Cryaa *gene, were created: the intact α*A-crystallin *148 kb BAC (αA-BAC) and αA-BAC(ΔDCR3), which lacks approximately 1.0 kb of genomic DNA including DCR3. Expression of EGFP in the majority of both BAC transgenics nearly recapitulated the endogenous expression pattern of the *Cryaa *gene in lens, but not outside of the lens. The number of cells expressing αA-crystallin in the lens pit was higher compared to the number of cells expressing EGFP. Next, we generated additional lines using a 15 kb fragment of α*A-crystallin *locus derived from αA-BAC(ΔDCR3), 15 kb *Cryaa/EGFP*. A 15 kb region of *Cryaa/EGFP *supported the expression pattern of EGFP also in the lens pit. However, co-localization studies of αA-crystallin and EGFP indicated that the number of cells that showed transgenic expression was higher compared to cells expressing αA-crystallin in the lens pit.

**Conclusion:**

We conclude that a 148 kb αA-BAC likely contains all of the regulatory regions required for αA-crystallin expression in the lens, but not in retina, spleen and thymus. In addition, while the 15 kb *Cryaa/EGFP *region also supported the expression of EGFP in the lens pit, expression in regions such as the hindbrain, indicate that additional genomic regions may play modulatory functions in regulating extralenticular αA-crystallin expression. Finally, deletion of DCR3 in either αA-BAC(ΔDCR3) or *Cryaa *(15 kb) transgenic mice result in EGFP expression patterns that are consistent with DCR's previously established role as a distal enhancer active in "late" primary lens fiber cells.

## Background

Gene regulation during embryonic development is regulated at the DNA level by a number of proximal (promoters) and distal (enhancers, silencers, insulators and locus control regions) regulatory regions that are organized by a variety of histone and non-histone proteins present in chromatin [1]. Ocular lens development has been extensively studied to address the molecular mechanisms that control tissue-specific, temporal and spatial gene regulation [2,3]. The mammalian lens is composed of two cell types, the anterior proliferating lens epithelial cells and the posterior differentiating lens fiber cells, both of a single progenitor origin. Lens fiber cells are characterized by a high concentration of crystallins, a family of proteins which constitute 90% of the water soluble proteins, and act as structural proteins in the lens [4-6]. These crystallins play key roles in maintaining lens transparency and in generating its refractive index. The relative simplicity of the lens embryonic origin, and its structure, make it an ideal model system to study the complexities of gene regulation [2,3].

Lens fiber cell differentiation is accompanied by the accumulation of high levels of αA-crystallin, representing 20–40% of the crystallin content of the human and mouse lens [4-6]. In addition to its role as a structural protein, αA-crystallin is also a member of the small heat shock protein family, thereby functioning as a molecular chaperone that prevents protein aggregation [7], and possesses an autokinase activity [8]. Disruption of α*A-crystallin *results in the formation of cataracts [9,10] (see MGI website for *Cryaa *phenotypic alleles), and has also been shown to result in apoptosis of lens epithelial cells [11], illustrating its vital role for lens homeostasis [9,10] (see MGI website for *Cryaa *phenotypic alleles). Furthermore, genetic studies in humans have shown that numerous αA-crystallin missense mutations result in cataract formation [10,12-19]. More recent studies have shown that αA-crystallin binds to and suppresses caspase 6 activity [20]. Although αA-crystallin is expressed predominantly in the lens, low levels of its expression have been reported in the retina, spleen and thymus [21,22]. However, no specific function of αA-crystallin has been shown in these tissues.

Our recent studies have identified two α*A-crystallin *lens-specific enhancers, DCR1 (located ~-8 kb) and DCR3 (located ~+3 kb, following the last Cryaa exon) [23] as novel distal regulatory regions involved in transcriptional regulation of the α*A-crystallin *gene. Conventional transgenic approaches utilizing DCR1 and DCR3 fused to the 1.9 kb αA-crystallin promoter were successful in expressing EGFP in both lens epithelium and fiber cells. However, while endogenous αA-crystallin expression was first apparent in the lens pit at E10.5, these transgenics expressed EGFP one day later in the lens vesicle, leading us to hypothesize that additional *cis *regulatory elements were required to recapitulate endogenous αA-crystallin expression. In this study we utilized BAC transgenics as a means to dissect further the transcriptional regulation of the α*A-crystallin *gene. Our main goal was to narrow down the genomic region(s) that harbour lens-specific essential regulatory regions required for the initiation of *αA-crystallin *gene expression in the lens pit.

## Results

### Endogenous αA-crystallin expression in mouse eye

**Figure 1:**
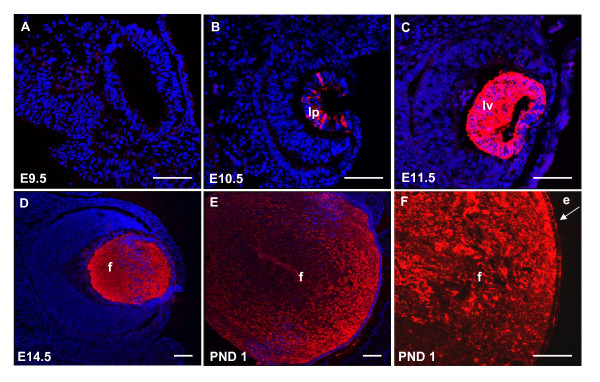
**αA-crystallin protein expression during lens morphogenesis**. Nuclear DAPI staining is blue and αA-crystallin immunolabeling is red. αA-crystallin is not expressed at E9.5 in the lens placode (A) but becomes visible in the lens pit at E10.5 (B). Expression intensifies in the developing lens vesicle at E11.5 (C), and is evident in the newly differentiated fiber cells at E14.5 (D), as well as within the lens epithelium. αA-crystallin expression continues in the fiber (E) and epithelial cells of the P1 lens (F). Lens epithelial cells, e; lens fiber cells, f; lens vesicle, lv; lens pit, lp. Scale bar = 100 μm.

Consistent with previous reports of αA-crystallin mRNA expression monitored by *in situ *hybridizations [24], endogenous αA-crystallin protein expression is first apparent in the lens pit at E10.5 (compare Fig. 1A with Fig. 1B). Expression is up-regulated at E11.5 in the lens vesicle (Fig. 1C) and continues to be highly expressed in the differentiating primary fiber cells at E14.5 (Fig. 1D), and within the fiber cells of the newborn lens (Fig 1E, F). Lower levels of αA-crystallin are expressed in lens epithelium compared to lens fiber cells of the postnatal day 1 (PND1) lens (Fig. 1F).

### Expression pattern of EGFP driven by 148 kb *Cryaa *BAC, αA-BAC

**Figure 2:**
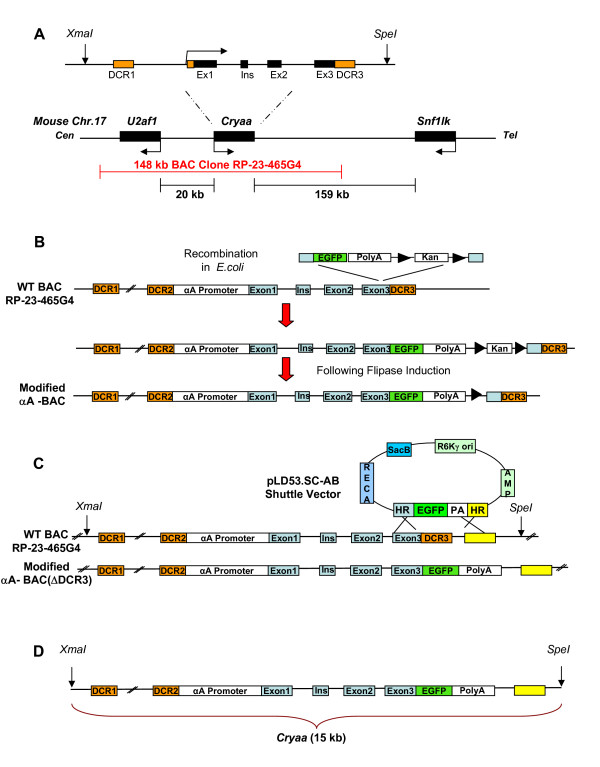
**Schematic representation of three αA-crystallin transgene constructs**. (A) A schematic diagram of the mouse *Cryaa *locus (chromosome 17) and its adjacent loci, *U2af1 *and *Snf1lk*. The 148 kb BAC Clone RP-23-465G4 is shown in red. The *Xma*I-*Spe*I sites delineate 15 kb of the *Cryaa *locus. Exons in the *Cryaa *gene (black box), DCR1 and DCR3 (orange box), centromere, cen; telomere, tel; Exon, ex; rodent specific *Cryaa *exon, Ins. (B) Modification of αA-BAC using the λ prophage system for homologous recombination [25]. (C) Generation of αA-BAC(ΔDCR3) using a shuttle vector [26]. (D) Diagrammatic representation of the 15 kb portion of the mouse αA-crystallin locus with an EGFP insert (see panel (C)) marked by unique *XmaI *and *SpeI *restriction sites.

Prior studies identified two distal control regions, DCR1 and DCR3 (Fig. 2A), that, in combination with a 1.9 kb promoter fragment, recapitulate most aspects of transcriptional regulation of αA-crystallin [23]. However, in these studies, the onset of EGFP expression was between E11-E11.5, which is at least 12 hours after the onset of endogenous αA-crystallin expression (see Fig. 1B). This finding suggests other regulatory regions could be required. To address this, we generated a BAC transgenic by modifying a 148 kb BAC encompassing the α*A-crystallin *gene (see Fig. 2A) through an in frame insertion of EGFP into the third exon, by homologous recombination according to [25] (see Fig. 2B). EGFP was inserted after V146, close to the C-end of the 173 amino acid αA-crystallin. Four founders were generated, but only three were studied due to breeding difficulties encountered with the fourth founder. All founders had visually green eyes, and transgene integrations were subsequently confirmed by genotyping. The copy number of the transgenic lines was determined by qPCR (Table 1) as described in Methods.

**Table 1:** Summary of transgenic mice used to study *Cryaa *expression in lens.

**Constructs**	**Regulatory ** **Elements**	**Copy Number ** **Range**	**Onset of ** **Expression**	**Expression ** **Location**
αA-BAC 4 lines*	148 kb BAC	2–3	E10.5 (3/3)	Lens (epithelium and fiber cells)
αA-BAC(ΔDCR3) 3 lines	148 kb BAC-DCR3 (with 1 kb deletion)	2–5	E10.5 (1/3)	Lens (epithelium and fiber cells)
Cryaa (15 kb) 6 lines/analyzed 4	15 kb Cryaa (-DCR3)	5–15	E10.5 (4/4)	Lens (epithelium and fiber cells) Brain

* One line had breeding issues and was not studied

**Figure 3:**
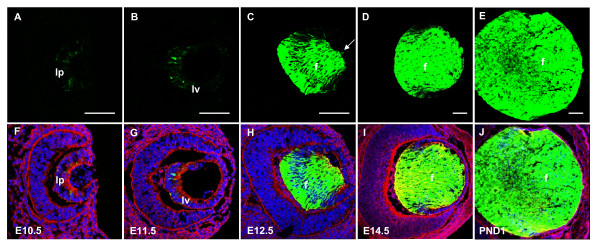
**Expression analysis of αA-BAC transgenic mice**. EGFP expression at E10.5 in the lens pit (A, F) and within the E11.5 lens vesicle (B, G). Expression is upregulated in lens fiber cells at E12.5, and very small expression is also apparent in a few cells of the lens epithelium (arrow) (C, H). Strong EGFP expression continues in the fiber cells at E14.5 (D, I) and in the PND1 lens (E, J). Nuclei are stained blue with DAPI, and the cytoskeletal staining is red. Lens fiber cells, f; lens pit, lp; lens vesicle, lv. Scale bar = 100 μm.

Embryonic EGFP expression analysis revealed that all three lines first express EGFP at E10.5 (Fig. 3A, F), which is temporally congruent with the onset of endogenous αA-crystallin expression (Fig. 1). Though EGFP is expressed in the lens vesicle at E11.5 (Fig. 3B, G), it becomes significantly upregulated at E12.5 in the differentiating primary lens fiber cells. Significantly lower EGFP expression was found in a small number of the overlying epithelial cells compared to the primary lens fiber cell compartment (Fig. 3C, H). This pattern of EGFP expression continues through E14.5 (Fig. 3D, I; see Additional file 1A for expression in lens epithelial cells) and in the PND1 lens (Fig. 3E, J; Additional file 1B), with high levels present in the differentiating lens fiber cells and lower expression evident in some cells of the lens epithelium. Thus, expression of EGFP inserted into the 148 kb *Cryaa *BAC (Fig. 2), due to its earlier onset, better recapitulates the expression patterns of endogenous αA-crystallin in lens (Fig. 1) when compared to the expression from DCR1/αA/EGFP transgenes [23].

### Removal of a 1.0 kb region containing DCR3 does not affect early αA-crystallin/EGFP expression in αA-BAC(ΔDCR3)

To test if DCR3 is essential for expression of *Cryaa *gene, we generated a BAC transgene which lacks approximately 1.0 kb of the *Cryaa *genomic region, including DCR3 downstream of the third exon (nucleotides +3,171 to +4,197) (see Fig. 2C). This results from a genomic deletion that occurs simultaneously with the insertion of EGPF into the third exon using the pLD53.SC-AB shuttle vector [26]. Three lines are generated which express EGFP and contain two to five copies of the transgene (see Table 1).

**Figure 4:**
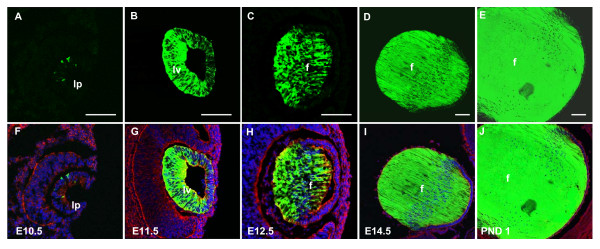
**αA-BAC(ΔDCR3) transgene expression in the lens**. Expression of EGFP from αA-BAC(ΔDCR3) is first observed at E10.5 in the lens pit (A, F). EGFP is expressed in the lens vesicle at E11.5 (Fig. B, G) and within the differentiating primary fiber cells at E12.5 (C, H), E14.5 (D, I) and in PND 1 lens (E, J). Fluorescent nuclear DAPI staining is blue and cytoskeletal staining is red. Lens fiber cells, f; lens pit, lp; lens vesicle, lv. Scale bar = 100 μm.

Similar to αA-BAC, EGFP expression is first evident in the lens pit at E10.5 (Fig. 4A, F) in one of the three αA-BAC (ΔDCR3) lines. Expression of EGFP is also detected in the lens vesicle (Fig. 4B, G) and is upregulated considerably in the differentiating lens fiber cells at E12.5 (Fig. 4C, H). As development progresses, EGFP is intensely expressed in the lens fiber cells at E14.5 (Fig. 4D, I; Additional file 1C) and PND 1 (Fig. 4E, J; Additional file 1D). Lower EGFP is also evident in some lens epithelial cells at these stages (Additional file 1C, D). The remaining two lines (see Table 1 and 2) have a delayed onset of EGFP expression, first found in the lens vesicle stage of development (data not shown).

**Table 2:** Relative expression of EGFP compared to αA-crystallin in transgenic lenses.

**Constructs**	**Regulatory Elements**	EGFP/αA RNA **
αA-BAC	148 kb BAC	1.3–47×
αA-BAC(ΔDCR3)	148 kb BAC – DCR3	1.2–6×
Cryaa (15 kb)	15 kb – DCR3	0.44–2.7×
pA425*	1.9 kb promoter + DCR1	0.456
pA427*	1.9 kb promoter + DCR1 + DCR3	0.094

*) Lines shown previously in [23].

**) Range between multiple lines analyzed.

### A 15 kb *αA-crystallin/EGFP *fragment which lacks DCR3 is sufficient for the earliest expression in the lens pit

The distance between the 5' end of DCR1 to the 3' border of DCR3 in mouse is approximately 13 kb and is marked by a corresponding domain of lens-specific chromatin [23]. In addition, our present (see Fig. 4) and earlier data [23] suggest that DCR3 may not be required for expression either in the lens pit or lens vesicle. To narrow down the genomic region required for EGFP expression in the lens pit, we released a 15 kb *XmaI-SpeI Cryaa/EGFP *fragment from αA-BAC (ΔDCR3) (see Fig. 2D) and used it to generate six transgenic founders, all of which expressed EGFP in the lens. The two lines showing the lowest expression of EGFP were not analyzed further.

**Figure 5:**
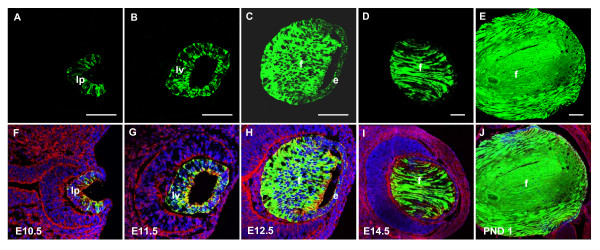
**Expression of EGFP from the 15 kb Cryaa/EGFP fragment in lens**. EGFP expression is first evident at E10.5 in the lens pit (A, F) and becomes expressed throughout the lens vesicle at E11.5 (B, G). As fiber cell differentiation commences at E12.5 (C, H), intense EGFP expression is observed. Prominent expression is also evident in the overlaying lens epithelium (C, H). EGFP expression continues to be highly expressed both in the lens fiber and epithelial cells of the developing E14.5 (D, I) and PND1 lens (E, J). Nuclear DAPI staining is blue, and the cytoskeletal phalloidin staining is red. Lens epithelial cells, e; lens fiber cells, f; lens pit, lp; lens vesicle, lv. Scale bar = 100 μm.

**Figure 6:**
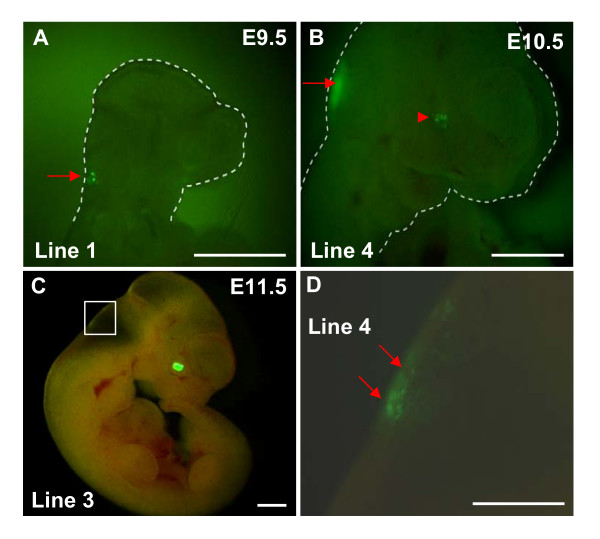
**Extralenticular expression of EGFP in 15 kb Cryaa/EGFP transgenics**. EGFP expression was observed at E9.5 (A), E10.5 (B), E11.5 (C) and (D) (higher magnification of (C)) (red arrows) in the hindbrain. The dashed white line illustrates the embryo and red arrowhead indicates EGFP expression in the lens. Scale bar = 1 mm.

All four remaining lines expressed EGFP at E10.5 in the invaginating lens pit (Fig. 5A, F). As development progressed, strong EGFP was found throughout the lens vesicle at E11.5 (Fig. 5B, G). In addition to the characteristic upregulation of EGFP in the differentiating fiber cells at E12.5, all transgenic lines also displayed intense EGFP expression in the lens epithelium (Fig. 5C, H), which was less evident in the *αA-crystallin *BAC transgenics (compare with Fig. 3C, H and Fig. 4C, H). Similarly, the lens epithelial cells at E14.5 (Fig. 5D, I; Additional file 1E) and at postnatal day 1 (Fig. 5E, J; Additional file 1F) also displayed stronger EGFP expression in the lens epithelium compared to their BAC counterparts. However, while high levels of EGFP expression were observed in lens, numerous embryos from different lines also exhibited extralenticular expression in regions such as the hindbrain, and other areas of the head (Fig. 6). From these results we conclude that expression of αA-crystallin/EGFP in the lens pit is regulated by one or more enhancers present presumably within the 15 kb *XmaI-SpeI *genomic fragment, functionally distinct from DCR3.

### Transgene expression of EGFP compared to endogenous αA-crystallin expression

**Figure 7:**
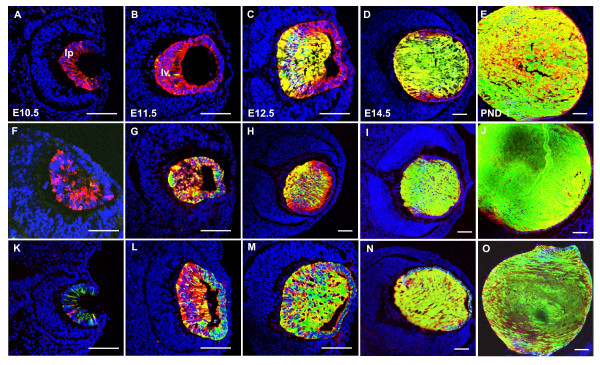
**Transgene expression coincides with αA-crystallin protein**. Panels A-E are sections from αA-BAC mice; F-J are from αA-BAC(ΔDCR3) mice; and K-O are from the 15 kb Cryaa/EGFP mice. Red staining is αA-crystallin immunofluorescence, green is EGFP fluorescence and yellow represents co-localization of these two signals. Scale bar = 100 μm.

Immunolabeling with anti-αA-crystallin antibody was conducted to evaluate co-expression with the transgenes visualized via EGFP. The representative data on αA-crystallin (red channel) and EGFP (green channel) proteins are shown in Fig. 7. A few yellow (merged red and green signals) cells in the lens pit of αA-BAC (Fig. 7A) and αA-BAC(ΔDCR3) (Fig. 7F) transgenics at E10.5 demonstrate co-expression of EGFP and αA-crystallin (red staining). However, while the expression of αA-crystallin seems to prevail over EGFP in the lens pit (E10.5) in both BAC models, it appears that transgene EGFP is catching up to endogenous αA-crystallin as more yellow signal is observed at E12.5 (Fig. 7C, H) compared to E11.5 (Fig. 7B, G). In contrast, co-expression analysis of the 15 kb Cryaa/EGFP unit at E10.5 (Fig. 7K), shows more cells with a stronger EGFP signal over the αA-crystallin.

At E11.5, while a small number of cells appeared to co-express αA-crystallin and EGFP in the lens vesicle of the αA-BAC transgenics (Fig. 7B, G), far more cells co-expressed apparently both proteins in αA-BAC(ΔDCR3) (Fig. 7G, see yellow cells) and in the 15 kb Cryaa/EGFP lenses (Fig. 7L). Coinciding with the onset of lens fiber cell differentiation at E12.5, lines from all three trangenes displayed significant co-expression of both proteins in lens primary fiber cells (Fig. 7C, H, M). At E14.5 (Fig. 7D, I, N) and postnatally (Fig. 7E, J, O), most lens fiber cells gave very strong EGFP signals over the red signals that originated from both the endogenous and fusion/EGFP proteins (see Discussion).

### Expression of EGFP in non-lenticular tissues of all transgenic models

EGFP expression was not microscopically observed in any non-lenticular embryonic tissue of αA-BAC or αA-BAC(ΔDCR3) transgenics. In order to determine whether EGFP was expressed in any specific non-lenticular tissue, we performed qRT-PCR analysis of total RNAs obtained from 1-day old lens, retina, brain, thymus, heart, spleen, kidney, liver and muscle. We did not detect any expression of EGFP mRNA by qRT-PCR (C_t _> 40, see Methods) in any non-lenticular tissue examined.

Extralenticular EGFP expression however was visually evident in the brain and head region of several of the 15 kb *Cryaa*/EGFP embryos from multiple lines (Fig. 6). Analyses by qRT-PCR of the Cryaa and EGFP mRNAs (see Methods) further substantiated exogenous expression in regions such as the brain, retina, kidney, spleen and muscle and their levels were found to be 0.3%, 0.4%, 0.04%, 0.02% and 0.08% as compared to EGFP expression in the lens, respectively. These results suggest that regions outside of this 15 kb/*EGFP *fragment, while present in the 148 kb *Cryaa *BAC, may play a role in modulation of extralenticular expression of α*A-crystallin*.

## Discussion

Initial studies of mouse α*A-crystallin *gene expression have shown that the αA-crystallin promoter fragment -366 to +46 supports expression of a linked CAT gene only in lens fibers [27,28]. Two novel regulatory regions of the mouse *Cryaa *locus, DCR1 and DCR3, have been recently identified and studied in transgenic mice [23]. Using the DCR1/1.9 kb promoter/EGFP reporter system, we detect initial EGFP expression in the lens vesicle (E11.5) although the onset of endogenous αA-crystallin commences in the invaginating lens placode/lens pit around E10.5 (Fig. 1). This finding suggests that other regulatory regions are required for the proper onset of αA-crystallin expression as the lens placode invaginates to form the lens pit (Fig. 1). Here, we find that a modified 14 kb region of the mouse *Cryaa *locus (a 15 kb transgene containing 14 kb of genomic *Cryaa *region with a 1.0 kb insert of *EGFP/polyA*) can support robust EGFP expression in the lens pit of E10.5 mouse embryos (Figs. 5A, F, and 7K), and, thus, represents a reasonable source from which to identify the "earliest" enhancer of the *Cryaa *locus. Moreover, the 148 kb *Cryaa *BAC is insufficient for appreciable EGFP expression in the retina, spleen and thymus [22].

### Novel insights into the temporal and spatial regulation of *αA-crystallin *gene expression in lens and extralenticular tissues

All three αA-BAC transgenic lines display both temporal and spatial expression patterns of EGFP that are qualitatively comparable to those of the endogenous αA-crystallin in lens. EGFP expression is first detected at E10.5 in the lens pit, and subsequently expressed in both the lens epithelium and fiber cells of the developing and newborn lens. The only significant difference between EGFP and αA-crystallin expression is much lower number of cells co-expressing both proteins between E10.5 and E11.5 (Fig. 7A, B). The most likely reason for this difference is the position-dependent distinct epigenetic regulatory mechanisms [1,29] between the endogenous *Cryaa *locus and randomly integrated 148 kb *Cryaa *BACs.

Previous studies conducted with traditional transgenics suggested that DCR3 plays a specific role during "late" primary lens fiber cell differentiation [23]. Herein, the deletion of DCR3 in both αA-BAC(ΔDCR3) and the 15 kb *Cryaa/EGFP *trangenes did not have any prevailing effect on the onset of EGFP expression in the lens, compared to the expression of αA-BAC transgenes, as 5 out of 7 independent lines (Table 1) showed EGFP expression in the lens pit. The number of lines analyzed here is comparable to similar transgenic studies [30-32]. Though DCR3 constituted a portion of the *Cryaa *3'-UTR, its deletion seemed not to affect the average expression of EGFP mRNA obtained from both BAC transgenes (Table 2). Nevertheless, the possible function of DCR3 in αA-crystallin mRNA processing and stability will require additional experimentation.

A lens-specific chromatin domain between DCR1 and DCR3 of the mouse *Cryaa *locus [23] may represent the minimal size of the mouse *Cryaa *locus (Fig. 2A). Since DCR3 appears to act as a "late" fiber-cell specific enhancer, we examined a 15 kb *Cryaa/EGFP *fragment without DCR3. From our results, it was evident that the genomic regions required for early temporal αA-crystallin expression in the developing lens are present within this 15 kb modified genomic fragment, as EGFP expression initiated in the lens pit at E10.5 in all four lines (Fig. 5). In fact, it appears that more cells in the lens pit expressed EGFP than endogenous αA-crystallin (Fig. 7K). This observation requires additional comments, as the αA-crystallin antibody is expected to recognize both αA-crystallin and αA/EGFP fusion proteins. For example, it is possible that the epitopes recognized by the polyclonal antiserum could be masked during the presumptive formation of αA-crystallin/EGFP oligomers [33]. It is also possible that the αA-crystallin/EGFP fusion proteins precipitate with other proteins to interfere with antibody recognition consistent with a range of lens opacities found in all transgenic lenses studied (see Additional file 2). Even if the protein remains unblocked, a constant, but unknown, fraction of the protein is bound by antibody under experimental conditions, whereas all of the transgene-derived protein is EGFP-tagged. Moreover, the relative fluorescence produced by each tag is different, so, although relative location can be estimated, relative amounts of two proteins within any sample should never be compared by this methodology. It is quite possible that the fluorescence intensity elicited from EGFP at sufficient concentration will always obscure the immunofluorescence signal from αA-crystallin under the experimental conditions used.

In the past, αA-crystallin was referred to either as a lens-specific, or as a lens-preferred protein with high expression in lens, and low expression in retina, spleen and thymus [3-5,12,13]. More recent high-throughput data, available through the MGI and Unigene websites (see Methods), expanded the previously established extralenticular regions of αA-crystallin expression to the heart, hindbrain, midbrain, cerebellum, pancreas, pituitary and a few other tissues. Thus, expression of EGFP driven by the 15 kb *Cryaa/EGFP *in regions such as the hindbrain from E10.5–14.5 (Fig. 6) may reflect its naturally low expression in this tissue. Analysis of guinea pig ζ-*crystallin *transcriptional control indeed identified a brain-specific regulatory region [34]. Interestingly, expression of EGFP, supported by the 148 kb *Cryaa *BAC, was not detected by qRT-PCR in spleen or thymus. One intriguing possibility is that expression in these tissues originates via elements shared with the adjacent *U2af1 *(expressed highly in thymus relative to other tissues such as the eye) and *Snf1lk *(expressed highly in spleen compared to other tissues) genes, but not present in the 148 kb BAC. A *U2 small nuclear ribonucleoprotein auxiliary factor gene*, *U2af1*, also carried in this BAC, encodes a heterodimeric splicing factor [35]. The αA-BAC transgenic lines also exhibit elevated U2af1 (data not shown), while expression of Snf1lk was not tested due to its absence in the 148 kb BAC (see Fig. 2A).

Thus, the present data suggest that the genomic regions required for αA-crystallin expression in the retina, spleen and thymus are not present in the 148 kb *Cryaa *BAC and may be located in genomic regions adjacent to this BAC clone, or, as in the case of genes such as olfactory receptors, these regulatory regions may be present on another chromosome [36]. The *interferon-γ *locus on chromosome 10 is similarly regulated by elements within a locus encoding the *Th2 cytokine *genes on chromosome 11 [30]. The complexity of the genome in relationship to the individual genes and their regulation is far more complex than previously thought [37,38].

### Transgene positional/copy number effects and lens structural integrity

Although the general concept is that large transgenes, such as BACs and YACs, are not susceptible to positional effects [31], the lack of EGFP expression at E10.5 in 2 out of 3 αA-BAC(ΔDCR3) transgenic lines suggests otherwise. Previous reports have indicated that generally one to five copes of BACs are inserted into the genome [32], however recent studies have suggested that this is not always the case. In fact, analyses conducted by Chandler et al., 2007 reveal that, while 50 % of transgenes are inserted as copies ranging between 1–5, BACs can be inserted with much greater copies, even up to almost 100 [32]. Additional BAC studies have reported copy numbers to be also in excess of 5 [39].

Analysis of the structural integrity of the adult lens reveals that both BAC and 15 kb *Cryaa/EGFP *transgenics display opacities (see Additional file 2). In fact, some of the αA-BAC and 15 kb *Cryaa/EGFP *transgenic mice begin to show signs of lens opacities upon eye opening (data not shown). Morphologically these adult transgenic mouse lenses appear to display nuclear cataracts, resembling human and mouse models of *Cryaa *cataracts [40]. Given the overpression of αA-crystallin/EGFP, one can speculate plausible mechanisms contributing to lens opacities. Firstly, it is possible that excess of αA-crystallin EGFP fusion proteins interferes with the stoichiometric ratio of αA and αB-crystallins in the α-crystallin heteroaggregate complex, leading to the misfolding and precipitation of proteins. Expression of the αA-crystallin/EGFP fusion protein may also play a role in disrupting other normal interactions of lens proteins, resulting in protein aggregation precipitation and cataract formation.

## Conclusion

The present studies have shown that a modified genomic fragment of 15 kb *Cryaa/EGFP *is sufficient to direct the earliest onset of αA-crystallin/EGFP expression in the lens pit (E10.5). The leading candidate regions to harbour this early enhancer activity are four exons (1–3 and ins), and evolutionarily conserved regions in introns 1 and 2 (see Fig. 2A).

## Methods

### Insertion of *EGFP *into 148 kb *Cryaa *BAC

The mouse α*A-crystallin *BAC clone RP-23-465G4 was obtained from screening the mouse RPCI 23 library as we described elsewhere [23]. αA-BAC was generated through a homologous recombination, according to Lee et al., 2001. Briefly, the BAC clone RP-23-465G4 was first transformed by electroporation into SW105 *E. coli *cells harbouring a defective λ prophage (kindly provided by Dr. Neil Copeland, NCI, Frederick, MD). A 2.4 kb linear targeting cassette containing EGFP-pA, FRT-Kan-FRT sequences was PCR amplified from pCS2+MTe-GFP-FRT-kan-FRT plasmid (kindly provided by Dr. Jim Lauderdale, University of Georgia, Athens, GA) with primers bearing 50 bp of homology to αA-crystallin and the plasmid. This targeting vector was PCR amplified with primers: 5'-CTCTCCTGCTCCCTGTCTGCGGATGGCATGCTGACCTTCTCTGGCCCCAAGGTCATGGTGAGCAAGGGCGAG-3' and 5'-CTCCCGTGACACAGGAATGGCCCTCTCGCTGTGGCCAGCATCCAAACCGGACTGTATTCCAGAAGTAGTGAG-3' using the following conditions: 95°C, 2 min for 1 cycle; 95°C, 30 sec; 60°C, 30 sec; 72°C, 2 min 30 sec for 30 cycles; 72°C, 7 min for 1 cycle. The PCR product was separated by gel electrophoresis followed by purification with the Qiaquick gel extraction kit (Qiagen) and the plasmid template removed by digestion with *DpnI*. Homologous recombination was conducted by electroporating 300 ng of the targeting vector into SW105 competent cells containing BAC clone RP-23-465G4, with a Bio-Rad gene pulser (1.75 kV, 25 μF, 200 Ω). Recombinants were verified by PCR using primers (5'-TTTGGCGCGCCAAGCCAGTTCCATACCCTGA-3' and 5'-CGGCGAGCTGCACGCTGCCGTCC-3'), and a single colony was used for FLP induction with 10% L (+) arabinose. Colonies were plated on chloramphenicol LB plates, and modified BAC's were verified by sequencing and restriction analysis.

The BAC clone RP-23-465G4 was modified, using the shuttle vector pLD53.SCAEB to insert EGFP into the third exon of α*A-crystallin*, to generate αA-BAC(ΔDCR3) transgenics [26]. Briefly, two regions homologous to α*A-crystallin *were PCR amplified using the following primers: Homologous region 1, 5'-TTTGGCGCGCCAAGCCAGTTCCATACCCTGA-3' and 5'-GTTTCTCCTCCCGTGACACA-3'; homologous region 2, 5'-TTAATTAAACACCACGGAACATACCACA-3' and 5'-CCGGCCGGGCTTGGACATCCAGGAACAG-3'. The homologous regions were cloned into the *AscI/SmaI *and *PacI/FseI *sites of the pLD53.SCAEB shuttle vector, respectively. 1 μg of the shuttle vector was electroporated into α*A-crystallin *BAC competent cells, using a Bio-rad gene pulser (1.8 kV, 25 μF, 200 Ω), and cointegrates were selected on LB plates containing chloramphenicol (20 μg/ml) and ampicillin (50 μg/ml). Cointegrates were analyzed by PCR with the following primers: 5'-GATGAGGAAGCTGGGTGGTA-3' and 5'-CGGCGAGCTGCACGCTGCCGTCC-3', and a correct integrate was subsequently resolved by growing in LB containing chloramphenicol and ampicillin and plated on LB + chloramphenicol + sucrose plates. Resolved colonies were analyzed by PCR with the following primers: 5'-CCTACGGCGTGCAGTGCTTCAGC-3' and 5'-CAAAGACAGCTCCATGCTGA-3', and modified clones were verified by sequencing.

### Isolation of a 15 kb *αA-crystallin/EGFP *fragment

The 15 kb α*A-crystallin/EGFP *construct was generated by digesting αA-BAC(ΔDCR3) with *XmaI *and *SpeI*. The digests were run on a 0.8% agarose gel (Seaplaque GTG) overnight, and stained with SYBR^® ^Gold (Molecular Probes). A 15 kb band was excised from the gel and electroeluted in TAE buffer. The DNA was precipitated with 100% ethanol and sodium acetate (pH 5.2; 300 mM), then washed with 70% ethanol. The 15 kb fragment containing 14 kb genomic DNA and 1.0 kb of EGFP/poly A was cloned into pBluescript SK II vector sites, *XmaI *and *SpeI*.

### Generation of transgenic mice

αA-BAC and αA-BAC(ΔDCR3) DNAs for pronuclear injection were isolated using the Qiagen endonuclease-free mega purification kit (Qiagen) according to the manufacturer's instructions, with the exception that the volume of P1, P2 and P3 was increased three times. Circular BACs and the the 15 kb genomic fragment DNA were injected into FVB/N fertilized oocytes (AECOM Transgenic and Gene Targeting Facility and NEI Genetic Engineering Facility). αA-BAC transgenic mice were identified by PCR of genomic mouse tail DNA with the following primers: 5'-CCTACGGCGTGCAGTGCTTCAGC-3' and 5'-GTTCTCCTCCCGTGACACA-3'. The primers used for αA-BAC(ΔDCR3) and the 15 kb locus transgenic lines were: 5'-TTTGGCGCGCCAAGCCAGTTCCATACCCTGA-3' and 5'-CGGCGAGCTGCACGCTGCCGTCC-3'.

### Immunofluorescence

Embryos were fixed in 4% paraformaldehyde, cryoprotected with 30 % sucrose in PBS, and embedded in tissue freezing medium™ (Triangle Biomedical Sciences) for cryosectioning. Transverse cryostat sections (6 μm) were collected, washed with PBS, and incubated for 30 minutes with Image iT™ FX signal enhancer (Molecular Probes). Slides were then washed in PBS and incubated overnight at 4°C with the primary antibody, αA-crystallin (1:1000) (sc-22743, Santa Cruz Biotechnology) diluted in PBS containing 1% BSA and 0.05% Triton-X100. Sections were washed twice for 10 minutes in PBS and incubated for 45 minutes with the secondary antibody, goat anti-rabbit Alexa Fluor^® ^568 (1:500) (Molecular Probes) and with DAPI (1:50,000) (Molecular Probes). Sections stained with rhodamine-phalloidin (1:300) (Molecular Probes) and DAPI (1:50,000) were incubated for 30 minutes at room temperature. Slides were washed with PBS and mounted with Vectashield (Vector). Images were taken with a Leica AOBS laser scanning confocal microscope.

### Quantitative RT-PCR (qRT-PCR)

RNA was isolated from dissected tissues with Trizol^® ^Reagent (Invitrogen) and digested with DNase I (Promega) according to manufacturer's instructions. cDNA was generated with Superscript™ III Reverse Transcriptase (Invitrogen), and the template was diluted 1:10. Primers used for qT-PCR were: αA-crystallin (5'-GAGATTCACGGCAAACACAA-3' and 5'-ACATTGGAAGGCAGACGGTA-3'), and EGFP (5'-ACGACGGCAACTACAAGACC-3' and 5'-GTCCTCCTTGAAGTCGATGC-3'; 5'-CACATGAAGCAGCACGACTT-3' and 5'-GGTCTTGTAGTTGCCGTCGT-3'). Primers for αA-crystallin recognize both endogenous and the fusion αA-crystallin-EGFP cDNA. The relative expression level of αA-crystallin was normalized by the EGFP fusion protein average versus endogenous αA-crystallin.

### Transgenic copy number analysis

Genomic DNA was isolated by digesting tissue with lysis buffer (100 mM Tris HCL, pH 8.0, 5 mM EDTA, 0.2 % SDS, 200 mM NaCl) containing Proteinase K (100 μg/ml) at 55°C. Phenol/chloroform/isoamyl alcohol (Invitrogen) extractions were performed, and DNA was precipitated with isopropanol. Quantitative PCR was conducted to determine the number of copies of the BAC transgene, using the following primers: αA-crystallin (5'-GAGAGGGCCATTCCTGTGT-3' and 5'-AGGGGACAACCAAGGTGAG-3'), (5'-GGGTGCTGGTCTACTTCCAG-3' and 5'-AACCACGACATCCGAAAAAG-3') and CCNI (5'-TCTTCTCCCTCCTCAGACG-3' and 5'-CCGTTACCACCTCATGATCC-3'); B2M (5'-CCCTGGCTGGCTCTCATT-3' and 5'-ACTGAAGCGACCGCGACT-3') for normalization.

### Bioinformatic searches

Mouse Genome Informatics (MGI) server  and Unigene EST Profile Viewer  were used to examine expression data of *Cryaa*, *Snf1lk *and *U2af1 *(see Fig. 2A) mouse genes. The MGI server also provides information regarding *Cryaa *phenotypic alleles .

### Supplementary methods

Mice lenses were dissected from animals ranging in age from 3–6 months. Opacities were demonstrated microscopically with an Olympus IX70 (Zeiss 1.25× NA 0.035) and pictures were taken with Cooke Sensicam Cooled CCD.

## Authors' contributions

LW, YY, EW and AC conceived the project. LW carried out all procedures resulting in modified BAC clones. YY participated in the data analysis and performed some qRT-PCR experiments. LW drafted the manuscript. All authors read and modified drafts and approved the final manuscript.

## Supplementary Material

Additional file 1 **Supplementary Figure 1. EGFP transgenic expression in the lens epithelium**. Figures (A, C, E) are E14.5 lenses and (B, D, F) are P1 lenses. Figures A) and B) show αA-BAC EGFP expression; C) and D) show expression of αA-BAC (ΔDCR3); and E) and F) show expression of 15 kb Cryaa in the lens epithelium. Red arrows indicate epithelial cells expressing EGFP. Scale bar = 100 μm. Click here for file

Additional file 2 **Supplementary Figure 2. Structural integrity and optical properties of the adult transgenic lens**. A wild type adult lens is shown in figure (A). Panels (B) and (C) illustrate αA-BAC (ΔDCR3), line 1 and line 2 lenses, respectively. EGFP expression in comparison to α A RNA was found to be approximately 6× for line 1 and 1.2× for line 2. Panels (D-F) represent lines 1–3 of αA-BAC, respectively. EGFP is expressed approximately 1.3, 37 and 47× in comparison to αA-crystallin expression in lines 1, 2 and 3, respectively. Figures (G-I) demonstrate opacities of Line 1, 2, and 3 15 kb lenses. Line 1, 2 and 3 express EGFP at approximately 0.44×, 2.7× and 0.5× to that of αA-crystallin. The degree of opacity in the lens seems to correlate with EGFP expression levels in all transgenic lines. Wild type, wt. Scale bar = 1 mm. Click here for file
