# Supplementary figures and images for: Transcriptional regulation of mouse alpha A-crystallin gene in a 148kb Cryaa BAC and its derivates
Source: BMC Dev Biol. 2008 Sep 19;8:88. doi: 10.1186/1471-213X-8-88 (PMC2567317; doi:10.1186/1471-213X-8-88)

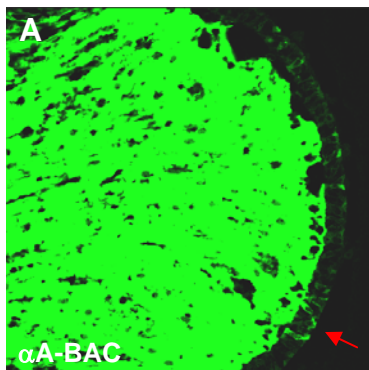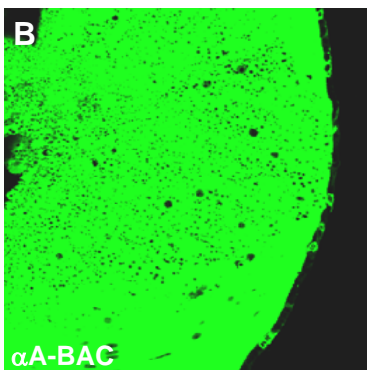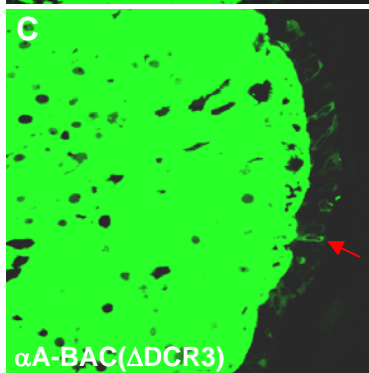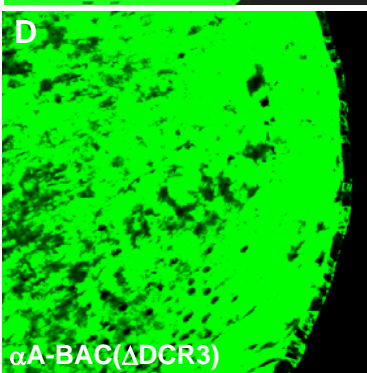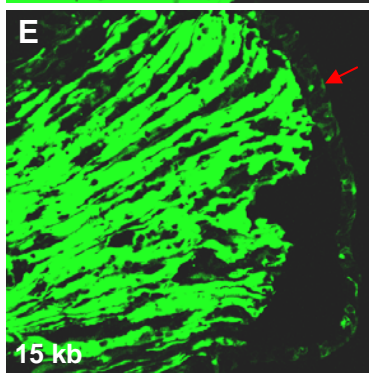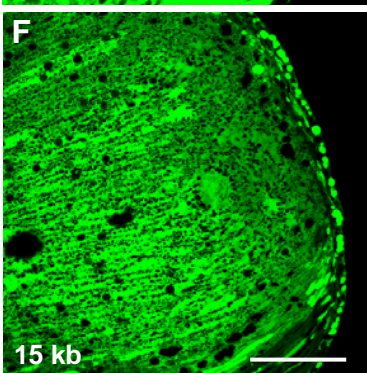

Supplement: Additional file 1 — Supplementary Figure 1. EGFP transgenic expression in the lens epithelium. Figures (A, C, E) are E14.5 lenses and (B, D, F) are P1 lenses. Figures A) and B) show αA-BAC EGFP expression; C) and D) show expression of αA-BAC (ΔDCR3); and E) and F) show expression of 15 kb Cryaa in the lens epithelium. Red arrows indicate epithelial cells expressing EGFP. Scale bar = 100 μm. [file 1471-213X-8-88-S1.pdf]

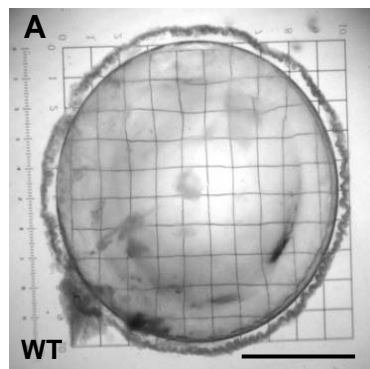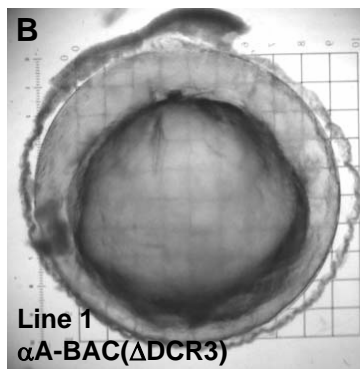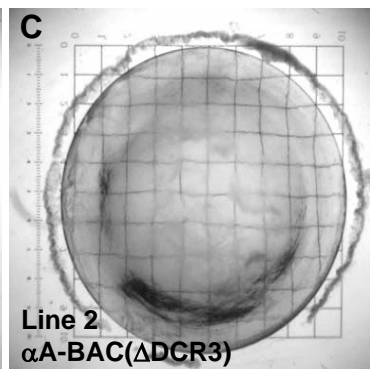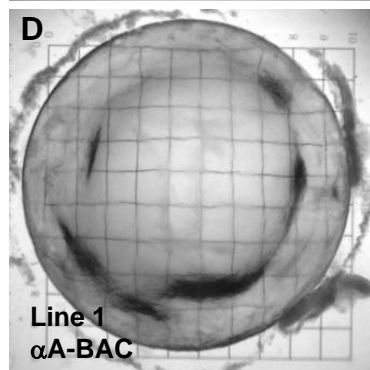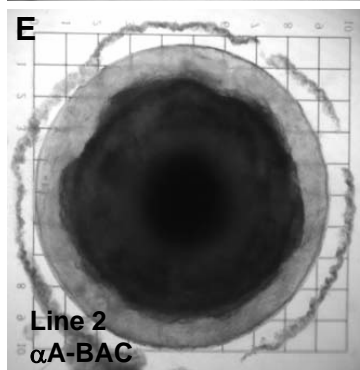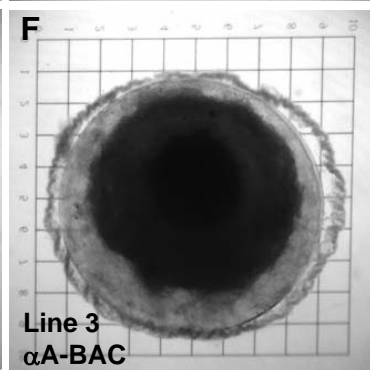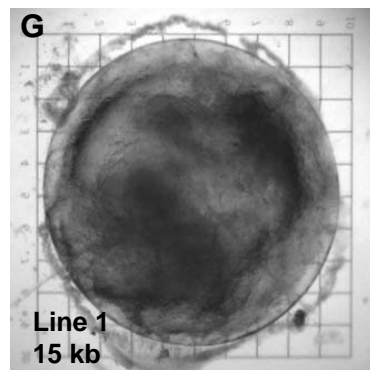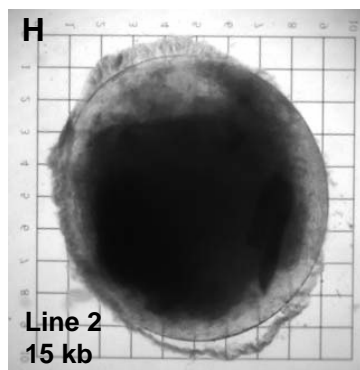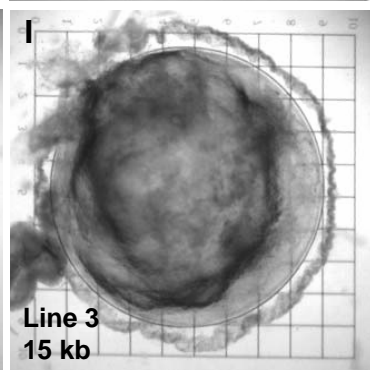

Supplement: Additional file 2 — Supplementary Figure 2. Structural integrity and optical properties of the adult transgenic lens. A wild type adult lens is shown in figure (A). Panels (B) and (C) illustrate αA-BAC (ΔDCR3), line 1 and line 2 lenses, respectively. EGFP expression in comparison to α A RNA was found to be approximately 6× for line 1 and 1.2× for line 2. Panels (D-F) represent lines 1–3 of αA-BAC, respectively. EGFP is expressed approximately 1.3, 37 and 47× in comparison to αA-crystallin expression in lines 1, 2 and 3, respectively. Figures (G-I) demonstrate opacities of Line 1, 2, and 3 15 kb lenses. Line 1, 2 and 3 express EGFP at approximately 0.44×, 2.7× and 0.5× to that of αA-crystallin. The degree of opacity in the lens seems to correlate with EGFP expression levels in all transgenic lines. Wild type, wt. Scale bar = 1 mm. [file 1471-213X-8-88-S2.pdf]
